# Reversion SAMD9 Mutations Modifying Phenotypic Expression of MIRAGE Syndrome and Allowing Inheritance in a Usually *de novo* Disorder

**DOI:** 10.3389/fendo.2019.00625

**Published:** 2019-09-11

**Authors:** Florence Roucher-Boulez, Delphine Mallet, Nicolas Chatron, Frédérique Dijoud, Daniela Brindusa Gorduza, Patricia Bretones, Yves Morel

**Affiliations:** ^1^Laboratoire de Biochimie et Biologie Moléculaire Grand Est, UM Pathologies Endocriniennes Rénales Musculaires et Mucoviscidose, Groupement Hospitalier Est, Hospices Civils de Lyon, Bron, France; ^2^Univ Lyon, Université Claude Bernard Lyon 1, Lyon, France; ^3^Centre de Référence Maladies Rares du Développement Génital: du Fœtus à l'Adulte, Filière Maladies Rares Endocriniennes, Bron, France; ^4^GReD, Université Clermont-Auvergne, CNRS UMR6293, INSERM U1103, Clermont-Ferrand, France; ^5^Laboratoire de Cytogénétique, Groupement Hospitalier Est, Hospices Civils de Lyon, Bron, France; ^6^Equipe GENDEV, CRNL, INSERM U1028 CNRS UMR5292, Bron, France; ^7^Laboratoire d'Anatomie Pathologique, Centre de Biologie et de Pathologie Est, Bron, France; ^8^Service Chirurgie et Urologie Pédiatrique, Groupement Hospitalier Est, Hospices Civils de Lyon, Bron, France; ^9^Service de Pédiatrie Endocrinologie, Groupement Hospitalier Est, Hospices Civils de Lyon, Bron, France

**Keywords:** MIRAGE syndrome, reversion mutations, revertant mutations, SAMD9, disorders of sex development, fetal growth retardation, small for gestational age

## Abstract

**Context:** MIRAGE (Myelodysplasia, Infection, Restriction of growth, Adrenal hypoplasia, Genital phenotypes, Enteropathy) syndrome is a severe multisystem disorder with high mortality. It is caused by a heterozygous gain of function mutation in the growth repressor gene *SAMD9*. The increasing number of reported cases displays a spectrum of phenotypes that may be explained by an adaptation mechanism, with appearance of a somatic second hit mutation with revertant effects.

**Objective:** To determine the genetic basis of the MIRAGE syndrome rapidly corrected in a living and healthy 46,XY patient.

**Subjects and Methods:** A 46,XY patient born with growth restriction and disorders of sex development had thrombocytopenia and necrotizing enterocolitis during the neonatal period suggestive of the syndrome. Faced with the rapid improvement of the patient's phenotype, an adaptation mechanism was sought by repeating genetic analysis at different ages; her parents also underwent genetic analysis.

**Results:** The previously described p.(Thr778Ile) mutation was identified and surprisingly transmitted by the asymptomatic mother in this usually *de novo* syndrome. To explain the rapid improvement of the patient's phenotype and absence of symptoms in the mother, an adaptation mechanism was sought. For the mother, a non-sense mutation was found (p.(Arg221^*^)) in *cis*, and most likely appeared *in utero*. It was not transmitted to her child. The child harbored a different non-sense mutation (p.(Arg285^*^)) that most likely appeared near day 20.

**Conclusions:** We show that pathogenic variants can be inherited from a healthy parent as the adaptation mechanism may arise early in life and mask symptoms. Presence of revertant mosaicism mutations could explain “incomplete penetrance” in other disease. For a better management and outcomes in patients, appearance of this natural gene therapy should be sought by repeating genetic analysis.

## Introduction

The recently described MIRAGE syndrome (MIM #617053) is the acronym of a severe multisystem disorder characterized by Myelodysplasia, Infection, Restriction of growth, Adrenal hypoplasia, atypical Genital phenotypes, and Enteropathy (1). Over the 35 patients reported in the literature with MIRAGE syndrome, variable phenotypic expressivity has been observed. Although the more consistent features seem to be the restriction of growth, adrenal hypoplasia, and disorders of sex development (DSD) in 46,XY patients, these symptoms may be missing (2, 3).

The syndrome is caused by a heterozygous gain of function mutation in a growth repressor gene located on chromosome arm 7q (*SAMD9)*, which mostly occurs *de novo*. The gain of function mutation increases SAMD9's effect as a negative regulator of cellular proliferation by altering the endosome system (1). To escape the inhibitory growth effect of the mutation, a compensator mechanism can take place with progressive loss of the mutated allele by monosomy 7, called “adaption by aneuploidy” and/or appearance of a somatic “second hit” within *SAMD9*, reversing the constitutional gain of function (1, 2). This adaptation mechanism rescues the life-threatening phenotype and explains the wide spectrum of the disease and survival of patients, but monosomy 7 increases the risk of myelodysplasia, which is only rescued by bone marrow transplant (4). Out of the 35 patients reported, 25 of them had an adaptation mechanism: 16 by “aneuploidy,” 9 by appearance of a loss of function mutation, and 5 with both mechanisms. Interestingly, important differences appear when looking at lifespan (1–9). The first patients identified were severely affected with a median lifespan of 10 months (1–9), while half of cases now reported are still alive (age ranges from 0.5 to 20 years) (1–9).

We report herein a new patient presenting a MIRAGE syndrome due to a variant inherited from her asymptomatic mother. Both the patient and her mother carry a distinct mosaic *SAMD9* loss of function mutation explaining the resistance toward the gain of function mutation, with improvement or absence of symptoms for the patient and the mother, respectively.

## Subjects and Methods

### Patient

**Figure 1:**
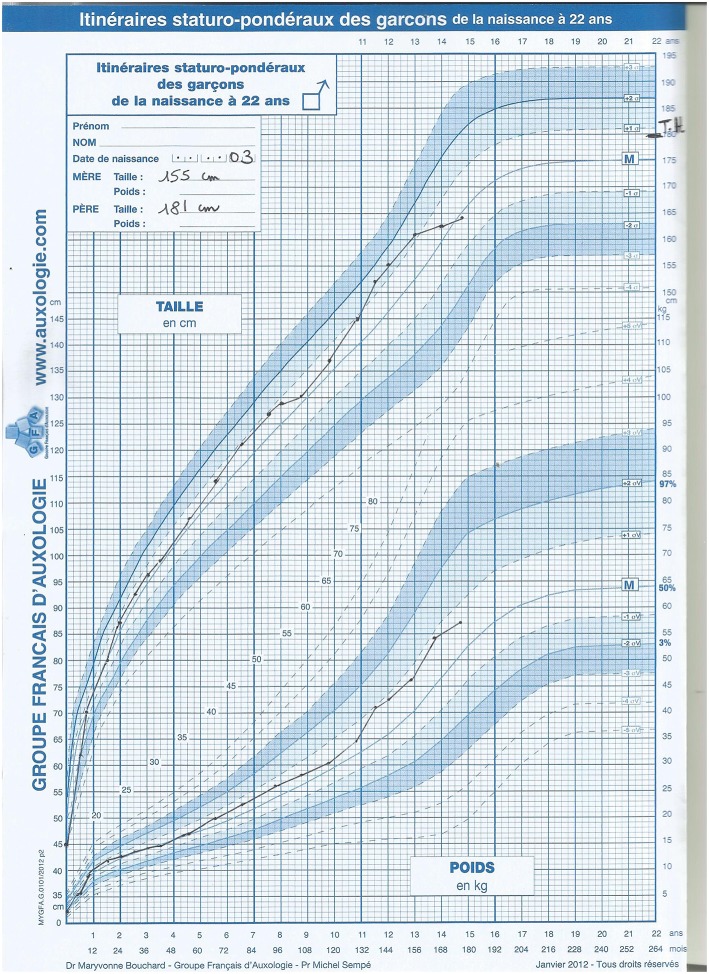
Patient's growth chart compared to the French reference Boy's growth chart.

The patient was born preterm (36 weeks and 5 days gestation) by emergency cesarean section for fetal distress with a good Apgar score and no respiratory distress, but intra-uterine growth restriction [IUGR; weight 1950 g (−2 SD), height 45 cm (−2 SD), head circumference 30.5 cm (−2 SD)]. At birth, the baby presented with severe hypoglycaemia resulting in a transfer to a neonatal unit for a single glucose infusion. There, the following disorder of sex development (DSD) was observed: very small, inguinal palpable testes, and a urogenital sinus (later during a urethroscopy it was revealed a blind ending vagina low implanted in the urethra). The genital tubercle had the appearance of a normal clitoris. The karyotype was 46,XY. Hormonal data at day 3 (D3) suggested gonadal dysgenesis and elimination of adrenal insufficiency: very low AMH 8 pmol/L (*N* > 200 pmol/L), undetectable testosterone, normal DHEAS (2952 nmol/L), and ACTH (20.9 ng/L). Severe thrombocytopenia (15,000/μL) at D3 was briefly corrected after platelet transfusion (D9: 126,000/μL; D10: 82,000/μL; D12: 76,000/μL) and followed by spontaneous progressive improvement (D16: 99,000/μL; D26: 165,000/μL; D42: 261,000/μL). At D7, the patient presented with necrotizing enterocolitis (occlusive syndrome with melena). The evolution of this enterocolitis was rapidly favorable within 12 days (D19). Retrospectively, the association of IUGR, 46,XY DSD, thrombocytopenia, and necrotizing enterocolitis suggested a MIRAGE syndrome. The new-born was reared as a girl and underwent feminizing genitoplasty at 6 months of age, as currently performed at that time. No Müllerian derivative was found during the laparotomy, and two very hypoplastic testes were removed. Microscopic histology found subnormal testicular tissue with abundant seminiferous tubules and germ cells. Rete testes were normal. During her follow-up for DSD, no adrenal insufficiency, even during surgery, and no myelodysplasia was observed. During the most recent examination, the 14-year-old patient had normal cognitive development, normal growth, and underwent puberty with hormonal substitutive treatment (without menstruations). However, the growth compared to the French reference Boy's growth chart shows the adult height will be 2 SDS below the target height for boys (Figure 1). Vaginal self-dilatation will be proposed upon her request later in life.

### Genetic Analysis

The SAMD9 single coding exon was sequenced using a classical Sanger method on DNA extracted from whole blood sampled from the patient at 5 days and 14 years of age old. Saliva was studied by targeted massively parallel sequencing at 14 years. The parents and the maternal grandmother also underwent genetic analysis. The maternal grandfather's DNA was not available. Written informed consents were provided by the parents of the patient, and the study was conducted in accordance with the principles of the Declaration of Helsinki and had Institutional Review Board approval. Sequence variants were designated using the NCBI reference sequences NM_017654.3 built on the GRCh37/hg19 reference genome. Pathogenicity of a variant was predicted using Alamut Genova^®^ Software (SOPHiA GENETICS, Saint Sulpice, VD, Switzerland). Array comparative genomic hybridization (aCGH) (SurePrint G3 Human CGH Microarray Kit, 4 × 180K, AMADID: 022060, Agilent Technologies, Santa Clara, CA, USA) was performed to identify a complete or partial monosomy 7 as a potential adaptation mechanism.

In order to determine whether the missense and non-sense mutations were in a cis or trans position, a fragment including both positions was amplified by long range PCR (3.1 kb) using a PrimeSTAR^®^ HS DNA Polymerase (Clontech Laboratories, Palo Alto, CA, USA). DNA fragments were inserted into the pCR-XL-2-TOPO vector using the TOPO XL-2 Complete PCR cloning kit (ThermoFisher, Carlsbad, CA, USA) according to the manufacturer's recommendations. Inserted fragments were sequenced using primers provided in the kit and internal primers used for SAMD9 sequencing.

## Results

The MIRAGE syndrome of the patient was explained by the variant c.2333C>T or p.(Thr778Ile).

This variant is not reported in any database to date (dbSNP, gnomAD, ClinVar…) but was reported by Schwartz et al. where they proved its cell cycle inhibiting effect (10). It affects an amino acid conserved between species, and the difference in chemical properties between the two amino acids is important (Grantham score: 89). *In silico* predictions are contradictory but usually suggest a loss of function mutation (considered deleterious by GVGD and SIFT software, benign by Polyphen-2 and MutationTaster). Furthermore, to assess the pathogenicity of a new variant in SAMD9, one could rely on its *de novo* appearance as many variants are present in population databases (1034 in gnomAD including around 700 missense mutations).

**Figure 2:**
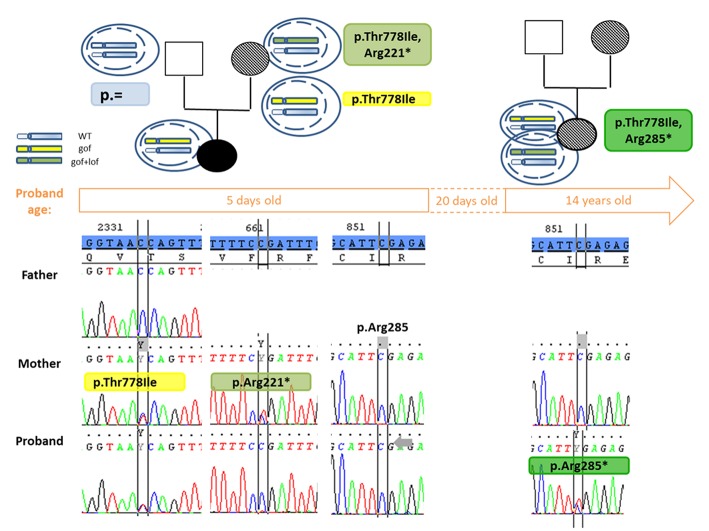
Pedigree and partial chromatograms showing the *SAMD9* mutations over time. The segregation of the gain of function (p.Thr778Ile) and the appearance of the reversion mutation (p.Arg221^*^, p.Arg285^*^) is shown over time. Above the time frame (corresponding to the age of the patient when DNA was sampled), next to the pedigree are shown a scheme of the haplotype in different cells: in blue is the wild type haplotype, in yellow the haplotype with a gain of function mutation and in green the haplotype with a gain of function disrupted by a loss of function mutation in *cis*. In the pedigree the symbol dashed indicates absence or correction of the symptoms found in MIRAGE syndrome. Under the time frame, partial Sanger chromatograms show presence or absence of the mutations at 5 days and 14 years of age for the proband. The reference sequence is highlighted in blue. At the 5 days old of the proband, the revertant mutation p.Arg221^*^ was found in *cis* in the mother but not transmitted to her children, confirming the adaptation mechanism in the mother occurred somatically and did not concern all tissues, at least the gonads. The correction of the symptoms around day 20 for the proband suggests the adaptive mechanism by appearance of the p.Arg285^*^ mutation was enough at this age, but it was only confirmed on a sample at 14 years old. WT, wild-type; gof, gain of function mutation; lof, loss of function mutation.

DNA sequencing of the parents, by targeting the mutation, found that it was inherited from the mother who had no documented history of MIRAGE syndrome. After re-questioning and checking the mother's health record, she indeed had growth restriction at birth: her weight was 1,580 grams and height 42 cm at the age of 37 SA. She did not have infection or enteropathy, but instead had anemia and coagulation disorders, which regressed spontaneously in the first month. No further medical problems appeared, and her adult height is 155 cm, the same as her mother. A reverted MIRAGE syndrome cannot be excluded. The maternal grandmother's DNA sequencing did not reveal presence of the mutation. Nevertheless, we hypothesized the mother had developed an adaptation mechanism, explaining the absence of symptoms. Sequencing the entire single exon confirmed the presence of another non-sense mutation: c.661C>T or p.(Arg221^*^) that was absent in the child's DNA (Figure 2). Topo cloning found the mutations in *cis* position (p.[(Thr778Ile; Arg221 = /Arg221^*^)];[ = ]). However, the proband did not carry this non-sense mutation. The improvement of her symptoms suggested that another adaptation mechanism had occurred near day 20. This was confirmed by identification of a second non-sense mutation: c.853C>T or p.(Arg285^*^) found in the second DNA sampled at 14 years of age (Figure 2); no monosomy 7 was identified at that time. Topo cloning also found the proband mutations to be in cis: (p.[(Thr778Ile;Arg285 = /Arg285^*^)];[ = ]). Saliva DNA analysis at 14 years of age for the patient and 38 years of age for the mother revealed the co-existence of the two changes. Targeted deep sequencing found a variant allele frequency of the non-sense mutation at 46 and 6%, respectively.

## Discussion

We herein report a 14 year old 46,XY DSD patient, currently living and raised as a girl, who presented with a MIRAGE syndrome rapidly corrected around day 20. Surprisingly, the SAMD9 variant, originally thought to be pathogenic, was inherited from the asymptomatic mother. To date, only one similar case has been reported, where a healthy parent transmitted the pathogenic SAMD9 allele to three offspring (6). The authors suggest a variable penetrance by mono-allelic expression However, to explain the phenotype improvement of our patient and the transmission by her healthy mother, we supposed that the already described adaptation mechanism had taken place for both subjects. In order to escape the inhibitory growth effect of the gain of function mutation, the organism adopts a survival strategy by progressively losing the mutated allele (by monosomy 7 or 7q), or by appearance of a somatic reversion loss of function mutation (1, 2), as a natural gene therapy with a growth advantage for the revertant cells. We found the child and the mother both had a somatic reversion loss of function mutations, p.(Arg285^*^) and p. (Arg221^*^), respectively.

How the adaptation mechanism occurs remains elusive. We suppose the mother's p.(Arg221^*^) had appeared *in utero*. The gain and loss of function mutations were found on the same allele in *cis* position, similar to the 4 other cases studied (6, 7). Since the mother's loss of function mutation was not transmitted to the child, it confirms that the adaptation mechanism is a post-zygotic event and does not concern all tissues, at least not the germ cells. We suppose the mother's p.(Arg221^*^) mutation appeared *in utero* at mid-gestation, after the end of ovarian germ cell division and the entrance of oogonia in meiosis. We suppose the reversion did not took place earlier in gestation because she had growth restriction at birth. For the child, the hypothesis is that the reversion took place in different tissues, at least in the haematopoietic system, near day 20 when the patient improved thrombocytopenia and enterocolitis.

The reversion may occur at different stages of development in different tissues, and an earlier adaptation in the adrenal gland could explain the absence of adrenal insufficiency, usually reported in the MIRAGE syndrome, found in this patient. The reversion could also be due to alternative mechanisms. In some patients, a co-existence of adaptation by aneuploidy (monosomy 7) and somatic second hit mutation are found (2, 6). Saliva samples were studied in the child and the mother and found coexistence of the two changes for both. As in cancer with somatic mutations, one could expect polyclonal patterns of adaptation, but it is striking that the same non-sense mutation found in blood cells was found in saliva. In the oral samples, leukocytes of mesodermal origin and squamous epithelial cells of ectodermal origin are found in a wide range of proportion. Furthermore, in adults with bone marrow allografts, the proportion of blood-derived from donor genetic material was shown to vary greatly in oral samples; indicating that haematopoietic cells can contribute to the DNA that is found in the oral cavity (11). However, a parallel can be made between the two paralogue genes *SAMD9* and *SAMD9L*, both residing on chromosome 7. These two genes were first associated with MIRAGE and Ataxia-Pancytopenia syndromes, respectively, but recent reports show an implication of germline mutations in pediatric myelodysplasia with a high propensity for monosomy 7. The same adaptation mechanisms are occurring for these two genes. For SAMD9L-related myelodysplastic syndrome, Pastor et al. hypothesized the possibility of hematopoietic stem cell niche repopulation by −7/del(7q), retaining only the wild-type SAMD9L allele to explain the recovery of complete blood counts and normalization of bone marrow cellularity for some patients (12). Similarly, for SAMD9, the spatial temporal events (adrenal gland, then haematopoietic system, gastrointestinal tract, saliva…) could be explained by a stem cell niche repopulation in which an initial somatic reversion mutation had occurred. The earlier the reversion occurs during embryogenesis, the more likely it is that revertant descendant stem cells spread as large clusters resulting in milder phenotypes (13). The time of the repopulation of a tissue by the revertant cells could depend on the vital role of the affected function. This needs to be proved by multi-organ sampling at different time points. It would explain the phenotypic expression modification found in the different cases reported.

There has been significant interest regarding somatic mutations in the causation of cancer, however their contribution to revert genetic events as a natural gene therapy is less described. Revertant mosaicism, where an inherited mutation is genetically corrected by a spontaneous event, has been discovered in an increasing number of heritable skin diseases, notably epidermolysis bullosa (13, 14). It was reported in keratitis-ichthyosis-deafness (KID) syndrome, which is caused by a heterozygous germline missense mutation in GJB2 gene. Ogawa et al. reported a healthy mother who had a heterozygous missense GJB2 mutation disrupted by a non-sense mutation and due to an allelic recombination. Notably, she transmitted only one mutation that elicits a congenital disease to her offspring (15).

In MIRAGE syndrome, the phenotypic expression seems to depend on this “natural protective second genetic event” and can lead to diagnostic difficulties. Some patients may miss core features of the syndrome, compensate for some features rapidly, or only have hematological symptoms (4, 6). This is highlighted in the patient reported herein, who is still alive and healthy, as well as in her asymptomatic mother. This phenomenon could be more frequent in diseases thought to be of “incomplete penetrance.” In a similar case where a healthy parent transmitted the pathogenic SAMD9 allele to three offspring, the authors suggest a variable penetrance. Again, if the comparison is made with SAMD9L, mutations are responsible for pediatric myelodysplasia transmitted by asymptomatic parents. Authors indicate that incomplete penetrance is noted but parental reversion second hit cannot be ruled out (6, 12). It could also explain the presence of clear pathogenic alleles for severe pediatric diseases in control databases such as gnomAD. One should be aware of this possibility, especially when looking at genes previously described in such phenomenon. This makes pathogenicity prediction difficult and can alter the predicted risks for future pregnancies.

Nevertheless, patients with MIRAGE syndrome require multidisciplinary monitoring as it can have a positive impact on their outcome. In the most severe forms, they usually have repetitive invasive infections associated with enteropathy, immunodeficiency, thrombopenia, and possible adrenal failure requiring hospitalization in a neonatal intensive care unit. With the hope that a “natural gene therapy mechanism” will occur, repeating genetic analysis and searching for the loss of a mutated allele by monosomy 7 and/or appearance of reversion mutations during follow-up is of great importance. Still, monosomy 7 predisposes to myelodysplasia that imposes a hematological follow-up and sometimes a bone marrow transplant whose success may be limited (2, 4, 16). Csillag et al. report a case of MIRAGE syndrome, caused by a *SAMD9* mutation. They performed FISH and SNP array analyses every 3 months showing the co-existence of cells with monosomy 7 and complete cytogenetic remission with uniparental disomy of the long arm of chromosome 7 (UPD7q) (8). It can be compared with the Pastor et al. study where they demonstrated in two cases that SAMD9L–related disease can be associated with transient −7, occurring as a one-time clonal event followed by somatic correction of haematopoiesis achieved by UPD7q with double wild-type SAMD9L (12).

However, there is no recommendation for a good frequency of sampling. Moreover, sampling can be difficult in these babies with multisystemic disorder and growth restriction. Still, it can help in the decision for patients in the palliative care unit, giving hope in a natural gene therapy and survival. Nevertheless, a threshold indicating that the adaptive mechanism is enough to survive does not exist. Reporting more follow ups of these patients would be beneficial for the pediatricians.

In conclusion, this report highlights (1) the disease-causing variants may be transmitted by an asymptomatic parent harboring a second genomic event which further complicates genetic counseling and (2) the importance and necessity of repeating genetic analysis to identify an adaptation mechanism that may improve symptoms and survival of MIRAGE syndrome patients. (3) As the syndrome may be incomplete, sequencing of the *SAMD9* gene should be performed in cases of growth restriction associated with adrenal insufficiency and/or 46,XY DSD, as well as in isolated myelodysplastic syndromes.

## Data Availability

The raw data supporting the conclusions of this manuscript are available on request. The more relevant parts are shown in Figure 2.

## Ethics Statement

The studies involving human participants were reviewed and approved by Comité d'éthique du CHU de Lyon. Written informed consent to participate in this study was provided by the participants' legal guardian/next of kin.

## Author Contributions

FR-B, DM, NC, and YM: study design and data analysis and interpretation. All authors: clinical work-up and manuscript approval. FR-B, DM, NC, DG, PB, and YM: manuscript preparation.

### Conflict of Interest Statement

The authors declare that the research was conducted in the absence of any commercial or financial relationships that could be construed as a potential conflict of interest.

## Abbreviations

MIRAGE: myelodysplasia, infection, restriction of growth, adrenal hypoplasia, genital phenotypes, enteropathy
DSD: disorder of sex development
IUGR: intra-uterine growth restriction.
